# Eosinophil Cytokines, Chemokines, and Growth Factors: Emerging Roles in Immunity

**DOI:** 10.3389/fimmu.2014.00570

**Published:** 2014-11-10

**Authors:** Francis Davoine, Paige Lacy

**Affiliations:** ^1^Pulmonary Research Group, Department of Medicine, University of Alberta, Edmonton, AB, Canada

**Keywords:** chemokines, growth factors, allergy, asthma, inflammation, helminths, cancer

## Abstract

Eosinophils derive from the bone marrow and circulate at low levels in the blood in healthy individuals. These granulated cells preferentially leave the circulation and marginate to tissues, where they are implicated in the regulation of innate and adaptive immunity. In diseases such as allergic inflammation, eosinophil numbers escalate markedly in the blood and tissues where inflammatory foci are located. Eosinophils possess a range of immunomodulatory factors that are released upon cell activation, including over 35 cytokines, growth factors, and chemokines. Unlike T and B cells, eosinophils can rapidly release cytokines within minutes in response to stimulation. While some cytokines are stored as pre-formed mediators in crystalloid granules and secretory vesicles, eosinophils are also capable of undergoing *de novo* synthesis and secretion of these immunological factors. Some of the molecular mechanisms that coordinate the final steps of cytokine secretion are hypothesized to involve binding of membrane fusion complexes comprised of soluble *N*-ethylmaleimide sensitive factor attachment protein receptors (SNAREs). These intracellular receptors regulate the release of granules and vesicles containing a range of secreted proteins, among which are cytokines and chemokines. Emerging evidence from both human and animal model-based research has suggested an active participation of eosinophils in several physiological/pathological processes such as immunomodulation and tissue remodeling. The observed eosinophil effector functions in health and disease implicate eosinophil cytokine secretion as a fundamental immunoregulatory process. The focus of this review is to describe the cytokines, growth factors, and chemokines that are elaborated by eosinophils, and to illustrate some of the intracellular events leading to the release of eosinophil-derived cytokines.

## Introduction

Eosinophils are granulocytic white blood cells that are rare in healthy individuals, but become elevated in both blood and tissue compartments in helminthic parasite infection as well as allergic inflammation, particularly in late-onset persistent eosinophilic asthma (1). Typically, the number of eosinophils generated from the bone marrow in healthy individuals are low, resulting in relatively few cells circulating systemically. Eosinophils that are produced in the healthy bone marrow predominantly home to the gut mucosa, where they may be involved in maintenance of homeostasis with gut microbiota (2). The numbers of blood and tissue eosinophils are markedly altered in a range of specific inflammatory and allergic responses, where eosinophils may be found in high densities in mucosal tissues. The recruitment of eosinophils is thought to be orchestrated by a complex series of events involving antigen-presenting cells (APCs), mast cells, T cells, B cells, along with their released cytokines as immune signals.

**Figure 1:**
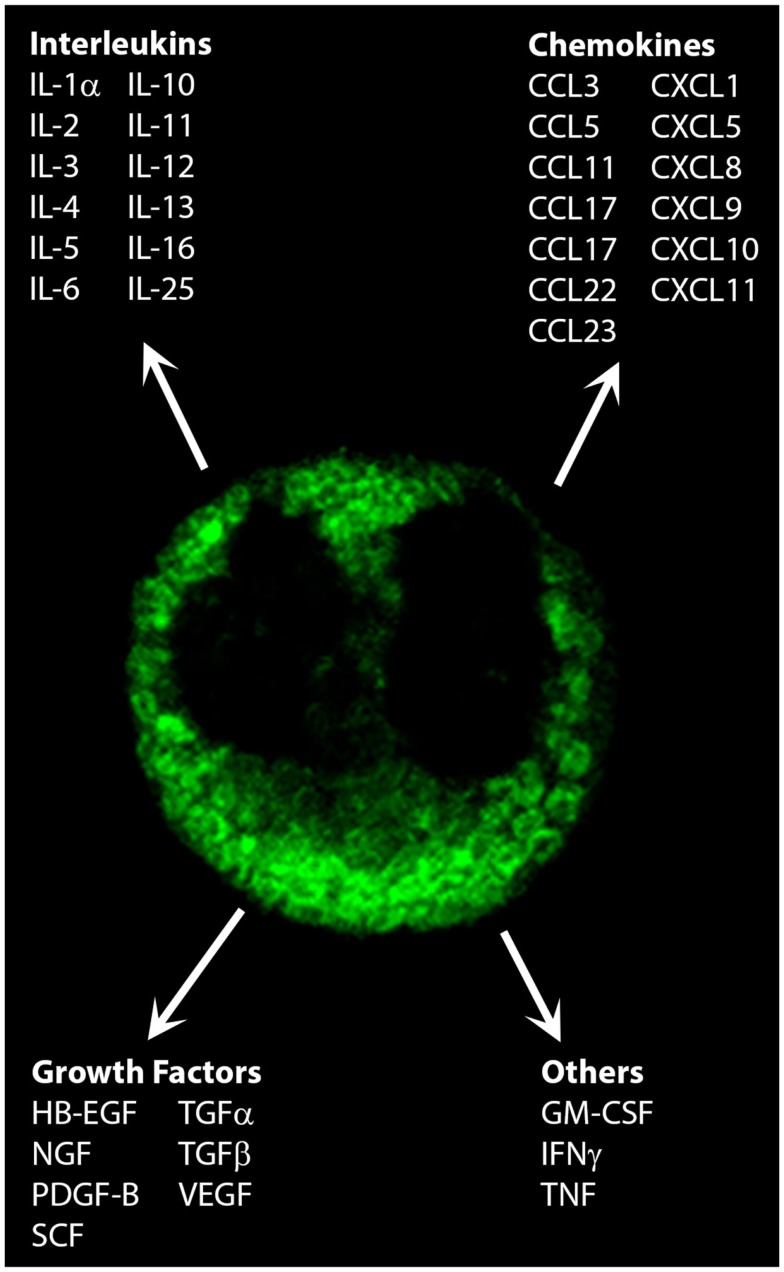
**Cytokines, chemokines, and growth factors secreted by eosinophils**. Shown is the immunofluorescent staining pattern obtained for CCL5/RANTES in a human peripheral blood eosinophil by confocal microscopy. Original magnification × 100.

Along with responding to immune signals, eosinophils themselves are a source of over 35 cytokines, chemokines, and growth factors (3, 4). These have profound effects on the progression of immune and inflammatory responses (Figure 1). The purpose of this review is to evaluate the role that eosinophil-derived cytokines, chemokines, and growth factors, and how these may contribute to the propagation of immune responses.

## Cytokines, Chemokines, and Growth Factors in Allergic Inflammation and Asthma

The role of eosinophils in immunity remains enigmatic. These granulated white blood cells are found, to varying degrees of similarity, in a wide range of invertebrates as well as vertebrates, including crustaceans, insects, mammals, fish, and birds (5, 6). Their expression in this wide variety of species suggests an important and evolutionarily conserved role in immunity. But what this role is, precisely, is still under intense scrutiny. While eosinophils have traditionally been implicated in maintenance of immunity against helminthic parasites, recent studies in transgenic mice that lack eosinophils suggest a more complex role for these cells than previously appreciated. In some cases, the absence of eosinophils actually inhibited parasitic growth (6), in contrast to the prevailing notion that eosinophils may be protective against helminthic parasites. More recent studies indicate that eosinophils may have a greater role in protection against viral infections, particularly respiratory viruses (7). Thus, the understanding of the role of eosinophils in immunity remains a fascinating and evolving area of research.

From a clinical perspective, eosinophils are widely known for their tendency to increase markedly during allergic inflammation in tissues that normally harbor very few eosinophils, such as the lungs and upper airways. A key feature of allergic inflammation and asthma is the acute or chronic inflammatory cell infiltration at sites of allergen exposure in atopic subjects. Eosinophils co-migrate with inflammatory cells and are thought to contribute to bronchoconstriction, mucus secretion, edema, and tissue injury in the airways. The inflammatory processes that underlie allergic responses are orchestrated by an elaborate network of cytokines and chemokines that regulate IgE responses, bone marrow progenitor cell differentiation, and adhesion molecule expression. Infiltration of inflammatory cells can further exacerbate inflammation in target tissues by further secretion of cytokines, chemokines, and growth factors from tissue-migrated cells. The allergic response is often manifested as a biphasic reaction in asthma, consisting of an early phase response that involves APC-mediated activation of T cells to a Th2 phenotype and mast cell degranulation, followed by the late-phase response in which a secondary infiltration of inflammatory cells occurs in affected sites. The role of eosinophils in the biphasic allergic response is thought to be mainly associated with the late-phase response. Eosinophils recruited to inflammatory sites frequently undergo degranulation, releasing a range of cationic cytotoxic molecules, including major basic protein (MBP) and eosinophil peroxidase (EPX), as well as producing numerous cytokines, chemokines, and growth factors.

Several regulatory cytokines have been defined as belonging to two classes of CD4^+^ T cells, which are involved in the initiation and maintenance of the allergic response. The first group of cytokines are those produced by T helper 1 (Th1) cells, which include interferon-γ (IFNγ), interleukin-2 (IL-2), and IL-12. The second group of cytokines are generated by Th2 cells, such as IL-4, IL-5, IL-9, and IL-13. More recent studies have suggested that Th17 and Treg cells are also important in the modulation of allergic responses by their production of immunosuppressive or regulatory cytokines including IL-10 and IL-17 (8). More recent studies have suggested an important role for IL-25 and IL-33 in the initiation of allergic responses, as these cytokines show a significant association with asthma in large cohort genome-wide association studies (9). In particular, IL-33 is important in the rapid induction of airway smooth muscle contraction by stimulating expansion of IL-13-producing type 2 innate lymphoid cells (10).

A substantial body of evidence from human and animal studies supports the hypothesis that allergic inflammation is an inappropriate response that arises from polarization of T cells toward a Th2 response, since greater expression of Th2 cytokines is seen in allergen-challenged individuals, along with a downregulation of Th1 cytokines. Enhanced expression of Th2 cytokines leads to the promotion of IgE switching of B cells, prevention of Th1 cytokine expression, increased tissue eosinophilia and eosinophil degranulation, and enhanced eosinophil survival. Prolongation of eosinophil survival, associated with delayed apoptosis, is thought to increase the amount of time that eosinophils actively release toxic mediators into tissues.

Against this background of Th2 responses in allergic inflammation, the cytokine network associated with allergy and asthma in humans is complex and not always associated with specific asthma phenotypes. Several studies suggest that although IL-4 triggers the polarization of T cells to a Th2 phenotype, it is not necessary for the manifestation of asthma (1). Moreover, the Th1 cytokine, IFNγ, may play a role in exacerbation of existing allergic inflammation as it is a potent activator of eosinophils *in vitro* (11–16). IFNγ has been found at elevated levels in the sera of patients with adult acute severe asthma (17, 18), and IFNγ^+^ cells become upregulated in correlation with eosinophil infiltration in allergic subjects (19, 20). Th1 and Th17 cytokines are associated with activation of innate immune cells in the recently characterized phenotype of non-Th2 asthma, which is a late-onset form of asthma that is seen in women, obese patients, smoking-associated asthma, and paucigranulocytic patients (1). Recent findings indicate that thymic stromal lymphopoietin (TSLP) may be a key target in airway hyperresponsiveness in allergic asthmatics (21). These observations suggest that Th2 cytokine responses alone are insufficient to promote asthmatic responses in the airways of human subjects.

However, the majority of asthma cases, although certainly not all, fit into the Th2 cytokine profile with varying degrees of eosinophilia (1). While the proportion of asthmatics exhibiting high numbers of eosinophils is not known, several studies of patients with mild to severe asthma suggest that it may be around 50% (1). Thus, eosinophils may be an important contributor to inflammatory responses at least half of asthma cases.

In summary, the substantial cytokine network underlying allergic inflammation is complex, with a Th2 cytokine profile and eosinophilia associating with some, but not all, asthma phenotypes. The way that eosinophil-derived cytokines contribute to immune defense or allergic diseases is not fully understood, although interestingly, recent discoveries have elucidated several novel functions for these cytokines in immunity and metabolism.

## Eosinophils and Their Degranulation Responses

**Figure 2:**
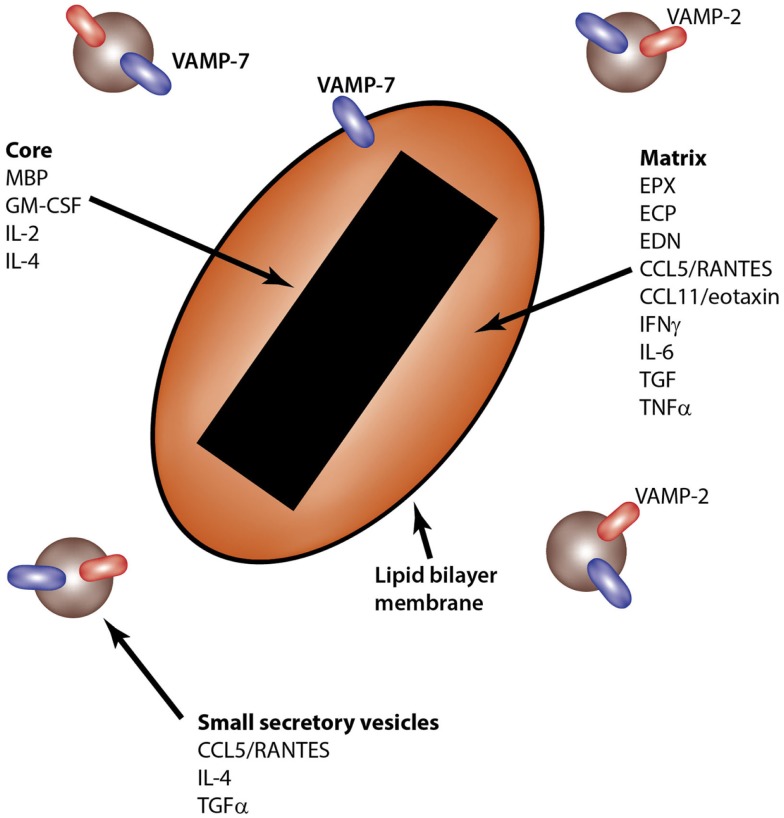
**Intragranular sites of storage for eosinophil-derived cytokines**. The eosinophil crystalloid granule consists of two internal compartments: the core, enriched in MBP, and the matrix, which contains EPX, ECP, and EDN, among other granule components. Small secretory vesicles also transport cytokines, including CCL5/RANTES, IL-4, and TGFα. SNARE molecules are shown in the lipid bilayer membranes of granules and secretory vesicles.

Eosinophils contain unique secretory granules known as crystalloid granules. These are so-called because of their characteristic crystalline cores, which appear electron-dense upon imaging by transmission electron microscopy. The crystalline core consists of highly concentrated, crystallized MBP, a cationic protein, which has cytotoxic effects on tissues upon its release (22). In addition to the MBP-rich crystalline core, crystalloid granules contain a matrix that is enriched in at least three other cationic proteins, which are EPX, eosinophil cationic protein (ECP), and eosinophil-derived neurotoxin (EDN). The liquid phase of the matrix also contains many other enzymes and proteins, including cytokines, chemokines, and growth factors (Figure 2).

The contents of the crystalloid granule in eosinophils are released to the outside of the cell by at least four distinct mechanisms. These are (1) classical exocytosis (23); (2) compound exocytosis (24); (3) piecemeal degranulation (25), which is a form of exocytosis involving the fusion of small, rapidly mobilized secretory vesicles with the cell membrane; and (4) necrotic disintegration of the cell or “cytolysis,” where whole, intact granules are released upon cell membrane rupture (26, 27). Piecemeal degranulation and cytolysis are most commonly observed in tissues obtained from patients with allergic inflammation (28, 29). Tissue damage associated with eosinophilic asthma and allergic inflammation is thought to be related to excessive release and tissue deposition of eosinophil granule proteins, particularly MBP, EPX, and ECP (22).

Several physiological agonists induce the release of eosinophil granule proteins by exocytosis, including platelet-activating factor [PAF; (30, 31)], opsonized surfaces (32), complement factors [C5a, (33)], immunoglobulin complexes (34), and cytokines and chemokines including granulocyte/macrophage colony-stimulating factor (GM-CSF), IFNγ, IL-3, IL-5, and CCL11/eotaxin (16, 35–37). Many of these factors are present in allergic inflammation and would be expected to contribute to activation of eosinophil degranulation responses.

## Human Eosinophils as a Source of Cytokines, Chemokines, and Growth Factors in Blood and Tissues

Over 35 cytokines, chemokines, and growth factors have been characterized in eosinophils (Table 1). In the majority of cases, messenger RNA and protein for each product has been identified. Evidence for the synthesis and expression of nearly all eosinophil-derived cytokines, chemokines, and growth factors has been obtained from peripheral blood eosinophils purified from non-atopic as well as atopic subjects. A number of these have been found as stored, pre-formed mediators in crystalloid granules, giving eosinophils the ability to release these potent immunoregulatory factors rapidly (<1 h) into the surrounding milieu in response to activation.

**Table 1:** **Eosinophil-derived cytokines, chemokines, and growth factors**.

Mediator detected in human eosinophils	Molecule detected	Mean quantity of protein stored (per 10^6^ cells)	Release factors	Intracellular localization of stored protein	Reference
**A. Cytokines**

A proliferation-inducing ligand (APRIL)	mRNA Protein	–	–	–	(38)

Granulocyte/macrophage colony-stimulating factor (GM-CSF)	mRNA Protein	15 pg	Ionomycin LPS	Crystalloid granules (core)	(39–42, 44)

Interleukin-1α	mRNA Protein	–	PMA	–	(45, 46)

Interleukin-1β	mRNA Protein	–	[Constitutively released]	–	(47)

Interleukin-2	mRNA Protein	6 pg	Serum-coated particles PHA	Crystalloid granules (core)	(48, 49)

Interleukin-3	mRNA Protein	–	Ionomycin IFNγ	–	(36, 40)

Interleukin-4	mRNA Protein	108 pg	Immune complexes Serum-coated particles Cytokines	Crystalloid granules (core)	(50–53)

Interleukin-5	mRNA Protein	–	Immune complexes	Crystalloid granules (core/matrix?)	(42, 54–57)

Interleukin-6	mRNA Protein	356 pg	Cytokines	Crystalloid granules (matrix)	(4, 15, 58, 59)

Interleukin-10	mRNA Protein	455 pg	Cytokines	Crystalloid granules	(4, 53, 60)

Interleukin-11	mRNA Protein	–	–	–	(61)

Interleukin-12	mRNA Protein	186 pg	Cytokines	Crystalloid granules	(4, 62)

Interleukin-13	mRNA Protein	4,596 pg	Cytokines	Crystalloid granules	(63, 64)

Interleukin-16	mRNA Protein	–	[Constitutively released]	–	(65)

Interleukin-17	Protein	–	–	–	(66)

Interleukin-25	mRNA Protein	–	[Constitutively released]	–	(67, 68)

Interferon-γ (IFNγ)	mRNA	997 pg	Cytokines	Crystalloid granules, small secretory vesicles	(4, 69)

Tumor necrosis factor-α (TNF)	mRNA Protein	909 pg	Immune complexes TNF LPS Cytokines	Crystalloid granules	(4, 43, 53, 70, 71, 72, 73)

**B. Chemokines**

CCL3/macrophage inflammatory protein-1α (MIP-1α)	mRNA Protein	–	–	–	(71, 74)

CCL5/regulated on activation, normal T cell expressed and secreted (RANTES)	mRNA Protein	7,000 pg	Serum-coated particles IFNγ	Crystalloid granules, small secretory vesicles	(16, 75)

CCL11/eotaxin	mRNA Protein	16–22 pg	C5a, immune complexes	Crystalloid granules	(76–78)

CCL13/monocyte chemoattractant protein-4 (MCP-4)	mRNA Protein	13 pg	Immune complexes	Crystalloid granules	(78)

CCL17/thymus activation regulated chemokine (TARC)	mRNA Protein	–	TNF + IFNγ or IL-4	–	(79, 80)

CCL22/macrophage-derived chemokine (MDC)	mRNA Protein	–	TNF + IFNγ or IL-4	–	(79, 80)

CCL23/myeloid progenitor inhibitory factor 1 (MPIF-1)	mRNA Protein	–	Cytokines	–	(81)

CXCL1/Groα	mRNA Protein	95 pg	Cytokines	Crystalloid granules	(82)

CXCL5/epithelial-derived neutrophil-activating peptide 78 (ENA-78)	mRNA Protein	1,500 pg	Cytokines	–	(83)

CXCL8/interleukin-8	mRNA Protein	–	C5a fMLP GM-CSF – RANTES or PAF Immune complexes TNFα LPS	–	(43, 53, 84–87)

CXCL9/monokine induced by gamma interferon (MIG)	mRNA Protein	–	TNF + IFNγ or IL-4	–	(79, 88)

CXCL10/interferon γ induced protein 10 (IP-10)	mRNA Protein	–	TNF + IFNγ or IL-4	–	(79, 88)

CXCL11/interferon-inducible T cell alpha chemoattractant (I-TAC)	mRNA Protein	–	IFNγ	–	(88)

**C. Growth factors**

Heparin-binding epidermal growth factor-like binding protein (HB-EGF-LBP)	mRNA	–	–	–	(89)

Nerve growth factor (NGF)	mRNA Protein	10 pg	–	–	(90)

Platelet-derived growth factor, B chain (PDGF-B)	mRNA	–	–	–	(91)

Stem cell factor (SCF)	mRNA Protein	9 pg	Chymase	Crystalloid granules	(92)

Transforming growth factor-α (TGFα)	mRNA Protein	–	Cytokines	–	(93–96)

Transforming growth factor-β (TGF-β)	mRNA Protein	–	Cytokines	–	(95–103)

Vascular endothelial growth factor (VEGF)	mRNA Protein	–	Cytokines	Crystalloid granules	(104–107)

In confirmation of observations with peripheral blood eosinophils, tissue eosinophils have also been characterized for their ability to synthesize and secrete cytokines, chemokines, and growth factors. Studies of tissue eosinophils from nasal polyps, bronchial biopsies, bronchoalveolar lavage (BAL) fluid, sputum samples, celiac mucosal biopsies, and skin biopsies from atopic individuals have shown that these cells are also capable of elaborating these immunomodulatory factors. For example, a significant percentage of eosinophils from subjects with allergic rhinitis express GM-CSF (41), IL-4 (50), IL-5 (108), CCL3/macrophage inflammatory protein-1α (MIP-1α) (71), CCL5/regulated on activation, normal T cell expressed and secreted (RANTES) (109, 110), and transforming growth factor-β1 (TGFβ1) (98). As many as 44% of eosinophils present in nasal polyp tissues have been shown to be positive for IL-4 (50). Moreover, the majority of tissue-infiltrating eosinophils (84%) were found to be IL-4^+^ during allergen-induced cutaneous late-phase reactions at 6 h (50). In another study, around 20% of tissue eosinophils were positive for IL-4 and IL-5 mRNA in skin biopsies of allergic individuals 24 h following challenge, which increased to 50–60% for protein expression of IL-4 and IL-5 (111). Eosinophils have also been shown to express IL-4 and IL-5 mRNA and protein in bronchial biopsies of atopic asthmatics as well as normal non-atopic subjects (112).

Other cytokines and chemokines have been shown to increase in tissue eosinophils during allergic inflammation. Eosinophils have been shown to exhibit greater expression of TGFβ1 than those of normal control subjects in bronchial biopsy tissue sections (101, 102). Nasal mucosal biopsies from seasonal rhinitis patients were found to contain elevated CCL5/RANTES^+^ eosinophils, making up around 15% of the total CCL5/RANTES^+^ population of cells (109). Eosinophils found in late-phase cutaneous reactions following allergen challenge in atopic subjects also express increased CCL5/RANTES mRNA and protein (75).

Endobronchial or segmental challenge with allergen consistently results in elevated eosinophil numbers in BAL fluid samples. Eosinophils accumulating in the airways following allergen challenge have been shown to express GM-CSF and IL-5 (42), as well as CXCL8/IL-8 (87), and CCL11/eotaxin (76). BAL-derived eosinophils that were recruited to the airways upon allergen challenge secrete significantly increased levels of CXCL8/IL-8 compared with those of normal controls during *in vitro* incubation (87). Supporting these observations is the study showing that sputum eosinophils also express GM-CSF as determined by immunocytochemistry (113).

The discovery of expression of cytokines by tissue eosinophils is not restricted to those found in the skin and airways. Eosinophils in the gut mucosa have also been found to express cytokines. In patients with active celiac disease, eosinophils from the gut mucosa were shown to be positive for IL-5 mRNA, and following treatment with a gluten-free diet, the numbers of IL-5^+^ eosinophils declined (54). However, IL-5^+^ eosinophils have not been detected in all gastrointestinal disorders, as intestinal mucosal eosinophils in Crohn’s disease do not appear to be positive for IL-5 (56).

Other diseases also exhibit cytokine expression by eosinophils. IL-5^+^ eosinophils have been detected in blood and tissue samples of individuals with eosinophilic cystitis, hypereosinophilic syndrome, and eosinophilic heart disease (55, 56).

Taken together, these and other *ex vivo* studies in humans have demonstrated that eosinophils derived from both the peripheral blood and tissue sources are capable of synthesizing, and in some cases releasing, cytokines, chemokines, and growth factors in eosinophilic diseases.

## Storage and Secretion of Eosinophil-Derived Cytokines, Chemokines, and Growth Factors

As many as 10 cytokines, chemokines, and growth factors have been identified as pre-formed mediators that are stored within the crystalloid granules of eosinophils. Those found within granules are CCL5/RANTES (16), CCL11/eotaxin (77), GM-CSF (44), IL-2 (48), IL-4 (51, 52), IL-5 (56, 57), IL-6 (15), IL-13 (4, 63), transforming growth factor-α (TGFα) (114), and tumor necrosis factor-α (TNF) (70). The most abundant cytokine in eosinophils appears to be IL-13, followed by IFNγ and TNF (4). The techniques used to determine intracellular sites of cytokine storage include immunocytochemistry, subcellular fractionation, immunogold labeling, and immunofluorescence using confocal microscopy analysis.

Most cytokines that have been identified in eosinophil crystalloid granules appear to be stored within the matrix compartment surrounding the crystalline core, although a few may colocalized with the MBP-containing core. These are GM-CSF (44), IL-2 (48), and IL-4 (51). Some cytokines have not had their precise intragranular location determined, such as IL-5. These fascinating observations indicate that eosinophils are capable of storing pre-formed cytokines that may be released rapidly in response to inflammatory events.

Eosinophils appear to use a specialized tubulovesicular system to transport some of these cytokines and chemokines from the crystalloid granule to the cell membrane. This membrane transport mechanism, also known as piecemeal degranulation, allows the shuttling of granule contents to the cell surface through rapidly mobilizable secretory vesicles that bud from the surface of the crystalloid granule (115–118). This mechanism of cytokine transport was first identified with CCL5/RANTES, in which at least two intracellular compartments store pre-formed cytokine. The first is the crystalloid granule, and the second is a pool of small secretory vesicles that sediment at a higher buoyant density than granules when analyzed by subcellular fractionation (16).

Other studies have demonstrated that these small secretory vesicles are important in cytokine trafficking, particularly for TGFα (114) and more recently, IL-4 (119–122). The membrane trafficking mechanisms associated with piecemeal degranulation of cytokines and chemokines are described in more detail elsewhere in this issue (123).

Small secretory vesicles increase in numbers as well as in their content of cytokines and chemokines upon stimulation of eosinophils by immunoregulatory cytokines. For example, stimulation of peripheral blood eosinophils *in vitro* by IFNγ induces intracellular mobilization of IL-6 and CCL5/RANTES prior to their release (15, 16). IFNγ specifically induces the redistribution of CCL5/RANTES within eosinophils from crystalloid granules to small secretory vesicles within 10 min of stimulation (16). Intriguingly, the pool of CCL5/RANTES-containing small secretory vesicles was mobilized to the cell periphery within minutes of stimulation, leaving MBP^+^ crystalloid granules behind in the cytoplasmic regions of cells, while crystalloid granule-associated CCL5/RANTES followed afterward. These findings suggest that granule proteins and cytokines/chemokines are selectively and differentially released in line with specific types of inflammatory responses in eosinophils.

Supernatants retrieved from IFNγ-stimulated eosinophils contained increased CCL5/RANTES and other crystalloid granule products (EPX), confirming the occurrence of degranulation (16). Following 16 h of stimulation by IFNγ, eosinophils were able to replenish their stores of CCL5/RANTES in their crystalloid granules. These findings have implications for sustained release of eosinophil-derived cytokines and chemokines in immune responses. An interesting possibility is that the eosinophil may have the potential to generate fine-tuned responses to immunological stimuli, with the release of cytokines/chemokines occurring separately from granule proteins.

CCL5/RANTES^+^ small secretory vesicles were subsequently found to colocalize with the soluble *N*-ethylmaleimide sensitive factor attachment protein receptor (SNARE), VAMP-2 (vesicle-associated membrane protein-2) (124). SNARE proteins are universal fusion proteins that regulate the attachment (docking) of lipid bilayer-surrounded granules or vesicles to target membranes such as the inner leaflet of the plasma membrane (125). The fusion of CCL5/RANTES^+^ vesicles upon docking with the inner leaflet of the plasma membrane is hypothesized to be dependent on binding to cognate target membrane-bound SNAREs, SNAP-23 and syntaxin-4 (117, 118, 126). Eosinophils depend on VAMP-7 for the release of granule proteins in response to intracellular secretagogues, GTPγS, and Ca^2+^ (127). However, we were unable to demonstrate whether CCL5/RANTES release was dependent on VAMP-7 as well, since negligible CCL5/RANTES was detected in supernatants of permeabilized eosinophils stimulated with these secretagogues. This suggests that the permeabilization process (using streptolysin-O) and/or the secretagogues used (GTPγS and Ca^2+^) may not be optimal for inducing piecemeal degranulation, leading to cytokine secretion, in eosinophils.

Our recent findings indicate that eosinophil secretion may be evoked by the addition of PAF, a potent secretagogue for both human and mouse eosinophils (31). Degranulation was assessed by an EPX ELISA that was optimized in-house (128). Previously, mouse eosinophils were considered to be poor degranulators (129–132). Using these novel parameters for the assessment of mouse eosinophil degranulation, we determined that the guanosine triphosphatases (GTPases) Rac2 and Rab27a contribute to the secretion of EPX from eosinophils (133, 134). Rac2 regulates the assembly of the actin cytoskeleton network, which is essential for granule movement through the cytoplasm, while Rab27a acts through a family of Sec/Munc proteins that regulate SNARE binding (135, 136). The role of GTPases in regulating cytokine secretion from eosinophils has not yet been defined.

Taken together, our studies suggest that cytokine trafficking and release in eosinophils may be mediated by VAMP-2 binding through its cognate SNAREs, SNAP-23, and syntaxin-4. Additional studies are required to understand how VAMP-2-mediated cytokine secretion may be regulated by GTPases.

## Potential Immunological Roles for Eosinophil-Derived Cytokines, Chemokines, and Growth Factors

The ability of eosinophils to synthesize and secrete a large number of cytokines, chemokines, and growth factors suggest that these cells have the potential to regulate numerous immune responses, including allergic inflammation. The diversity of immunomodulators produced by eosinophils suggests that they may be able to orchestrate inflammatory processes in an exacerbating or modulatory manner. Many of the factors elaborated by eosinophils are likely to regulate immune responses, particularly CCL3/MIP-1α, CCL5/RANTES, CCL11/eotaxin, CXCL8/IL-8, GM-CSF, IL-1α, IL-2, IL-3, IL-4, IL-5, IL-6, IL-9, IL-10, IL-13, TNF, and various growth factors. Other eosinophil-derived cytokines, chemokines, and growth factors, shown in Table 1, are likely involved in other types of reactions that apply to unique situations, such as atopic dermatitis, which may involve IL-12 released from tissue eosinophils following an initial phase of a Th2 response during cutaneous allergen challenge.

Eosinophil-derived chemokines may support the recruitment and maintenance of tissue eosinophils and lymphocyte infiltration during allergic inflammation. Eosinophils generate numerous chemokines including CCL3/MIP-1α and CCL5/RANTES (16, 71, 74, 75), both of which are major regulators of local inflammatory responses and chemoattractants for circulating leukocytes (137, 138). CCL5/RANTES exerts direct effects on eosinophils by elevating intracellular Ca^2+^, triggering degranulation, and promoting superoxide release concurrently with enhanced chemotaxis (139–141).

CCL11/eotaxin is an important eosinophil-specific chemokine that is involved in chemotaxis of eosinophils into tissues, and is a highly potent agonist for inducing an influx of eosinophils during allergic responses (142). Gene knockout of CCL11/eotaxin markedly diminishes the tissue presence of eosinophils, which subsequently decreases allergic inflammation in the gut, skin, and airways (143). Like CCL5/RANTES, CCL11/eotaxin activates intracellular Ca^2+^ mobilization, degranulation, and respiratory burst in eosinophils, suggesting that it may act in an autocrine manner (144–146). Eosinophils express CCL11/eotaxin constitutively, apparently in association with their granules (77). This suggests a potential role for eosinophil-derived CCL11/eotaxin to function in a paracrine/autocrine manner for further recruitment of eosinophils at sites of inflammation.

Eosinophils release the neutrophil chemokine CXCL8/IL-8 (84), suggesting a role for eosinophils in recruitment of neutrophils at sites of inflammation. This chemokine is highly chemotactic and stimulatory for neutrophils and T cells (147), and eosinophils also have chemotactic responses to CXCL8/IL-8 following incubation with GM-CSF or IL-3 (148). However, IL-4 and IL-5 production in allergic inflammation may downregulate CXCL8/IL-8 expression, since these cytokines inhibit CXCL8/IL-8 release from monocytes (149). The overall role of CXCL8/IL-8 derived from eosinophils in inflammatory conditions is yet to be determined.

Eosinophils have been shown to synthesize and release abundant GM-CSF, which promotes degranulation and mediator release from these cells (37, 40, 44, 148). The activation of eosinophils by ionomycin, a calcium ionophore, induces GM-CSF release, which prolongs their own survival *in vitro* (40). Thus, GM-CSF is likely a critical eosinophil-derived cytokine that is important in maintaining the viability and effector function of eosinophils at inflammatory foci in allergic and immune responses.

The production of IL-1α from human eosinophils has been associated with cytokine induction of human leukocyte antigen DR (HLA-DR) expression (45). This suggests that the eosinophil has the capacity to function as an APC, as demonstrated in mouse models of allergic inflammation (150–153). Eosinophils have also been demonstrated to function as APCs during *Strongyloides stercoralis* infection (154). Secretion of IL-1α from eosinophils may be an important factor in allergen presentation to T cells in subjects with established eosinophilia during Th2-deviated immune responses to specific allergens.

A role in allergic inflammation is also implicated for eosinophil-derived IL-2, an essential growth factor for T cells that is likely to be critical for the initiation of the allergic phenotype following an early phase involving IL-4 stimulation (155). IL-2 also induces eosinophil chemotaxis via its receptor (CD25) expressed on a proportion of them (156).

IL-3 is a pluripotent growth factor required for the generation of a wide range of myelocytic cells and granulocytes, and is an important autocrine factor produced and used by eosinophils when stimulated (148, 157). Thus, tissue eosinophils that are actively secreting IL-3 are likely to prolong their own survival by autocrine signaling.

The role of IL-4 in allergic inflammation has been extensively studied and is among the most clearly defined of all known cytokines, with biologics development targeting the function of this cytokine (158). This cytokine is firstly important in the maintenance of Th2 responses (159, 160), and secondly, it is a critical factor in initiating the switch of B cells to IgE isotype production (161). IL-4 also has many other stimulatory roles in allergic inflammation, by inducing chemotaxis in eosinophils and enhancing the capacity of eosinophils to release granule proteins (35, 162). Eosinophil-derived IL-4 may be important in promotion of inflammation by increasing local IgE production, as well as upregulating vascular cell adhesion molecule (VCAM) expression on endothelial cells, which would increase leukocyte adhesion and transmigration into affected tissues (163). This would, in turn, increase eosinophil-specific migration into tissues by expression of very late antigen 4 (VLA-4) ligand (164).

Similarly to IL-4, IL-5 is also extensively investigated and well-defined for its role in allergic inflammation. While IL-5 is not necessary for skewing immune responses toward a Th2 phenotype, it is important in the downstream events that are typical of Th2 responses to allergens. IL-5 is essential for the terminal differentiation of eosinophils from CD34^+^ progenitors present in the bone marrow (165, 166). It also has numerous effects on eosinophils including prolongation of survival, induction of chemotaxis, priming, and degranulation so that their responses to agonists are enhanced (35, 167, 168). Finally, IL-5 prolongs the survival of eosinophils *in vitro* (169). These observations suggest that eosinophil-derived IL-5 is involved in exacerbations of the local allergic response following its release.

An additional cytokine derived from eosinophils that is likely to contribute to allergic responses is IL-6. This pleiotropic cytokine, generated during acute phase reactions, is important in regulating T and B cell function, as well as priming of granulocytes. IL-6 is an essential cofactor with IL-4 in isotype switching of B cells toward IgE production (170). In asthmatics, IL-6 is elevated in the serum and BAL in both baseline conditions and following allergen challenge along with IL-4 and IL-5 (171, 172). Whether IL-6 expressed from eosinophils is important in allergic inflammation is not known.

Eosinophils may also augment Th2 responses by secretion of IL-9, a potent T cell, and mast cell growth factor (173, 174). The expression of IL-9 mRNA and protein products were demonstrated in eosinophils along with mRNA encoding the IL-9 receptor α subunit, suggesting an autocrine role for this cytokine in eosinophils.

Eosinophils have the capacity to express and release the immunosuppressive cytokine IL-10 (53). A role for eosinophil-derived IL-10 is likely to enhance allergic inflammation, since this cytokine acts in concert with IL-4 to mediate the growth, differentiation, and isotype switching of activated B cells (175). However, IL-10 is classically known for its immunosuppressive role in immunity by decreasing cytokine secretion from inflammatory cells and preventing allergic inflammation (176, 177). A recent study showed that eosinophil-derived IL-10 has a novel immunoregulatory role for eosinophils in helminth infection (60). IL-10 generated from eosinophils induced the proliferation of myeloid dendritic cells and CD4^+^ T lymphocytes, which inhibited expression of inducible nitric oxide synthase (iNOS), and protected intracellular *Trichinella spiralis* larvae. This striking observation suggests a protective role for eosinophils for intracellular *T. spiralis* larvae against NO-mediated killing, and further, that IL-10 derived from eosinophils drives this protective response. Further, it appears that *T. spiralis* exploits eosinophils to maintain its long-term survival in muscle tissue. These findings indicate a significant functional diversity of eosinophils that has not previously been appreciated until the advent of transgenic eosinophil-deficient mouse strains.

Among the more important cytokines released by eosinophils is IL-13, which is also stored in crystalloid granules as a pre-formed mediator (4, 63). IL-13 has many roles in the establishment of airway disease in asthma as well as pulmonary fibrosis, and also activates matrix metalloproteases in the airways (178). The activation of matrix metalloproteases by IL-13 is thought to protect against excessive allergic inflammation. IL-13 is also able to induce isotype switching of B cells to produce IgE (179), and has an important role in allergic inflammation (180). The expulsion of helminthic parasites from the gut of mice is also dependent on IL-13 (181). Eosinophils express IL-13 in inflammatory diseases (64), and this may have a role in the development of allergic inflammation, as discussed below.

Growth factors have an important role in inflammatory conditions, and those derived from eosinophils are likely to promote an inflammatory phenotype. Among these are heparin-binding epidermal growth factor (HB-EGF), which is a potent smooth muscle cell mitogen, and may contribute to pulmonary hypertension (89). Nerve growth factor (NGF) is elevated in subjects with allergic asthma, allergic rhinitis, and allergic urticarial–angioedema, with the largest increases observed in asthma (182). Eosinophils express NGF mRNA and protein, and this may contribute to the elevated levels seen in allergic inflammation (90). The most likely source of elevated serum NGF in allergy is from IgE-stimulated mast cells in tissues, since mast cells synthesize and secrete NGF (183). NGF stimulates T cells, B cell proliferation and differentiation, and eosinophil differentiation from peripheral progenitors (184), and is thus implicated in the pathogenesis of allergic inflammation.

Eosinophils also express the mast cell cytokine stem cell factor (SCF) (92). SCF may be associated with a positive feedback loop in tissue mast cells to maintain or exacerbate allergic inflammation, as well as inducing the growth and differentiation of mast cell progenitors residing in tissues (185).

Other growth factors produced by eosinophils include TGFα and TGFβ, which may modulate wound-healing and tissue remodeling (93, 102). TGFβ is specifically recognized for its role in chronic inflammation and fibrosis, and eosinophil-derived TGFβ may exert a role in tissue repair by inducing fibroblast growth and differentiation into myofibroblasts (186). Thus, TGFβ released from eosinophils may have a role in extracellular matrix protein deposition, particularly collagen, which contributes to structural abnormalities observed in severe allergic inflammation, including stromal fibrosis and basement membrane thickening.

Purified peripheral blood eosinophils from atopic individuals have been demonstrated to spontaneously release the pro-inflammatory cytokine TNF upon culture, and normal eosinophils stimulated with immobilized immunoglobulins or TNF express mRNA for this cytokine (53). TNF is a highly potent activator of monocytes, T cells, neutrophils, and endothelial cells, and enhances eosinophil adhesion and cytotoxicity (187, 188). This particular cytokine has numerous roles in inflammatory conditions, as well as helminthic infection and neoplasia associated with eosinophilic infiltration.

The Th1 cytokines, IL-12 and IFNγ, are also expressed in eosinophils. These are typically associated with inflammatory conditions that are distinct from the Th2 profile in allergic inflammation, and usually downregulate allergic inflammation following their release (189). However, Th2 cytokines can paradoxically promote Th1 responses in immune cells. IL-12 produced from eosinophils treated with IL-4 has been demonstrated to promote IFNγ mRNA expression in human Th1 cells (62). Allergen patch test reactions in atopic patients have shown that a Th1-like T cell activation occurs following the initial phase of increased local expression of Th2 cytokines associated with eosinophil infiltration after allergen challenge (190). Eosinophil-derived IL-12 was proposed to induce a switch from Th2 to Th1 responses commonly seen in late-phase allergic skin reactions (191). Eosinophils also express IFNγ in normal and atopic individuals (4, 69). Stimulation of eosinophils by IFNγ has been postulated to enhance allergic inflammation during viral infections, suggesting an autocrine role for this cytokine (192).

Taken together, these findings show that eosinophils have the capacity to generate numerous immunoregulatory cytokines, chemokines, and growth factors. However, while the discovery that eosinophils can synthesize and secrete these immunomodulatory factors is important, it is essential to determine whether eosinophil-derived cytokines, chemokines, and growth factors have bioactive roles in immunity. In this way, we may learn more about the specific immunological function of eosinophils in the regulation of inflammatory processes.

## Do Eosinophil-Derived Cytokines, Chemokines, and Growth Factors have Bioactive Roles in the Immune System?

Although the studies described above have presented evidence for the expression and release of a plethora of cytokines, chemokines, and growth factors from eosinophils, around a third of these have been shown to have a direct bioactive role. These include a proliferation-inducing ligand (APRIL), CCL5/RANTES, GM-CSF, IL-1β, IL-4, IL-10, IL-12, IL-13, IL-16, TNF, and TGFβ, which have been shown to have direct bioactive effects on other cells or in mouse models.

Recent studies indicate that eosinophils express APRIL, along with IL-6, which promoted the survival of plasma cells in the bone marrow in mice (38). This important finding suggests that eosinophils may have a role in enhancing and maintaining immunoglobulin production from plasma cells, which was shown to be true in a later study where eosinophils increased IgA^+^ plasma cell numbers and the secretion of IgA in the gut mucosa (193). These studies suggest that eosinophils are essential for maintaining the integrity of the gut mucosal immunity, which is in agreement with their usual tissue homing under homeostatic conditions.

Other studies have shown that eosinophil-derived CCL5/RANTES and IL-16 have direct effects on lymphocytes in culture by inducing chemotactic activities (62). Thus, human eosinophils have the ability to alter the function of CD4^+^ lymphocytes and memory T cells (194, 195). Finally, eosinophil-derived CCL5/RANTES has been shown to exert bioactive effects in eosinophil chemotactic assays as determined by the inhibitory effects of antibody to CCL5/RANTES, suggesting a role for this chemokine in autocrine signaling to enhance eosinophil migration.

The prolongation of eosinophil survival by GM-CSF is sensitive to inhibition by cyclosporin A, suggesting that this drug may modulate allergic inflammation by preventing autocrine cytokine signaling in eosinophils (40). Thus, eosinophil-derived GM-CSF is likely to have biological effects on survival of tissue eosinophils as well as newly recruited cells.

A recent study has demonstrated a role for eosinophil-derived IL-1β in inducing synthesis and secretion of IL-17 in activated CD4^+^ T cells (47). As Th17 cells are implicated in the pathogenesis of allergic airway inflammation (196), this may be a key mechanism by which eosinophils promote Th17 cell differentiation.

Perhaps the most well studied cytokine elaborated by eosinophils is IL-4. Eosinophils are among the most abundant IL-4-expressing non-T, non-B cell populations in the lung and spleen of mice infected with the helminthic parasite *Nippostrongylus brasiliensis* (197). In the IL-4 reporter 4get mice, eosinophils were the most prevalent IL-4-producing cells infiltrating the lungs of mice infected with *N. brasiliensis*, with an up to 1000-fold increase (198). IL-4 expression in eosinophils is constitutive and programmed at an early stage of ontogeny (199). Moreover, instillation of IL-4 led to expansion of IL-4-producing eosinophils *in vivo*, suggesting that IL-4 is a potent factor in promoting the differentiation of bone marrow progenitor cells into Th2 cytokine-producing eosinophils, using mice expressing IL-4 with GFP (4get) (200). The production of IL-4 by eosinophils also occurs in other infections; when mice are infected with the fungal *Cryptococcus neoformans*, the majority of cells expressing IL-4 in the airways are eosinophils (201).

Adjuvant stimulation of B cell responses has been linked to IL-4 production from eosinophils in the spleen, based on findings in Δdbl-GATA eosinophil-deficient mice that were administered with alum (153). When alum was injected intraperitoneally into eosinophil-deficient animals, early B cell priming and IgM production was attenuated. This suggests a pivotal and previously unrecognized role for eosinophils in modulating the adaptive immune response to vaccines.

Interestingly, recent findings have indicated that eosinophil-derived IL-4 is also essential for the biogenesis of beige fat, a type of brown adipose tissue that is found in abundance in newborns that promotes non-shivering thermogenesis (202). Eosinophil-derived IL-4 was demonstrated to switch monocytes to the alternatively activated macrophage phenotype (203, 204), leading to the conversion of beige fat precursor cells to beige adipocytes (205). In a separate study, the satiety hormone leptin was found to be a potent secretagogue for eosinophils, and induced the expression and secretion of IL-1β, IL-6, and CXCL8/IL-8, as well as other chemokines, from eosinophils (206). This observation suggests that eosinophils may have an important role in improving the metabolic phenotype, by promoting insulin responsiveness and decreasing the incidence of obesity.

Eosinophil-derived IL-4 may be important in liver regeneration by promoting hepatocyte proliferation (207). In this study, liver injury induced by partial hepatectomy or carbon tetrachloride (CCl_4_) in 4get mice resulted in the rapid recruitment of eosinophils, which secreted IL-4 and induced the proliferation of hepatocytes and liver growth.

The secretion of IL-4 from eosinophils in local tissues during allergic reactions may serve as a major initial source of IL-4 required for switching tissue-infiltrating naïve T cells to the Th2 phenotype. Several mouse models of Th2 inflammation support this notion. Mice infected intraperitoneally with eggs from the trematode *Schistosoma mansoni*, which generates a strong Th2 phenotype, exhibited early IL-4 increases derived from degranulating peritoneal eosinophils. Eosinophil-derived IL-4 induced priming of naïve T cells and activation of mast cell IL-5 release (154). The significance of IL-4 generation from eosinophils in human studies is yet to be discovered.

Stimulation of eosinophils with the Th2 cytokine IL-4 can also promote the development of Th1 responses. As described in Section “Potential Immunological Roles for Eosinophil-Derived Cytokines, Chemokines, and Growth Factors” above, IL-4-treated eosinophils release the Th1 cytokine IL-12, which in turn induced the expression of IFNγ from Th1 cells during the culture of T cells with eosinophil-conditioned media (62). The finding that eosinophils may release Th1 cytokines suggests a more nuanced control of cytokine responses in allergic inflammation that these cells may possess. In summary, IL-4 production from eosinophils may be important in a variety of immune and metabolic responses that are only just beginning to be understood. These observations will continue to shape our understanding of the biological role of eosinophils in immunity.

A fascinating recent study using a computational model demonstrated that eosinophil-derived IL-13 is required for allergic airway responses (208). This was determined by the use of IL-13^−/−^ eosinophils that were adoptively transferred by intravenous injection into Δdbl-GATA mice, which exhibited low airway hyperresponsiveness. IL-13 is important in inducing many of the characteristics of allergic airway disease, including airway hyperresponsiveness, goblet cell hyperplasia, and mucus secretion (180). In the computational model, it was found that eosinophil-derived IL-13 could not sustain an allergic asthma response in the absence of T cell (or other cell type)-derived IL-13, but that IL-13 production by eosinophils was integral to the development of allergic asthma (208).

Co-culture of human eosinophils and their conditioned media with the colon carcinoma cell line Colo-205 led to the production of TNF, which was involved in tumor cell killing along with granzyme A (73). This suggests a novel function for eosinophil-derived TNF in regulating tumor cell growth, and that tumor cells may be directly recognized by eosinophils.

Eosinophil-derived TGFβ has been shown to regulate fibroblast proliferation and differentiation (209). This is indicative of a potential role for eosinophils in wound-healing. Indeed, eosinophils were found to express both TGFβ and IL-13 following intradermal allergen challenge, which resulted in increased repair and remodeling events in human atopic skin (210).

## Other Physiological or Pathological Roles for Eosinophil-Derived Cytokines

Although earlier studies in mice suggested that eosinophil-derived IL-4 was required for mounting a Th2 response during immune reactions to intraperitoneally injected *Schistosoma mansoni* eggs (211), more recent studies have shown that ablation of eosinophils from mice had negligible outcomes, or even inhibitory effects, on helminth larval or egg expulsion in Δdbl-GATA or PHIL mice (212). These experimental findings imply that eosinophil-derived cytokines, chemokines, and growth factors have no specific role in the regulation of parasitic worm diseases, in contrast to the classically held notion that eosinophils are important in parasitic worm expulsion.

In other studies, certain types of lymphoid and solid tumors have been associated with the infiltration of eosinophils into cancerous tissues (213), particularly specific lymphomas and Hodgkin’s disease. Although tumor-related eosinophilia was considered to be an epiphenomenon arising from the spontaneous elaboration of IL-5 from tumor cells, or overproduction of T cells during chemotherapy with IL-2 (214), there is evidence of eosinophil activation as a result of IL-2 anticancer therapy (215, 216). In some cases, tissue eosinophilia is considered to be a positive prognosis for head and neck cancers (217) and advanced bladder cancer (218). Specifically, in oral squamous cancer, eosinophils are a positive prognosis for early stages of disease (Stages II and III), but an unfavorable prognosis for advanced cases (Stages III–IV) (219, 220). The involvement of eosinophil-derived cytokines, chemokines, and growth factors in neoplasias associated with eosinophilic infiltration is partially understood. In oral squamous cell carcinoma with tumor-associated tissue eosinophilia (TATE), the source of CCL11/eotaxin is apparently eosinophils (221). The cultured oral squamous cell carcinoma cell line SCC9 secretes chemotactic prostaglandin D_2_, which promotes eosinophil transmigration, thus providing a signal to recruit eosinophils into tumor masses (222). Eosinophils that infiltrate into tumors/lymphomas also express IL-6, TGFβ, and CCL24/eotaxin-2 (223).

However, while numerous studies have highlighted a role for eosinophil recruitment and activation in many types of cancers, and that eosinophils express receptors and mediators shared with cytotoxic T cells (224), there are few that implicate eosinophil-derived cytokines, chemokines, and growth factors in the regulation of cancer growth. Interestingly, as described above, a recent study showed that human eosinophils possessed tumoricidal activity toward a colon cancerous cell lines in culture by releasing TNF (73). The tumor-killing effects of eosinophil-derived TNF were the first description of a cytokine, chemokine, or growth factor elaborated by eosinophils implicating a role for these immunomodulators in cancer.

Injection of IL-17E increases the efficiency of chemotherapy and results in eosinophilia (225). Eosinophil-derived IL-17 may be implicated in antitumor activity, since IL-17E (IL-25) exerts antitumor activity against many types of cancerous cell lines, at least in xenograft models using human cancer cells in mice (226). Eosinophils express IL-17 as determined by immunocytochemistry and Western blot analysis (66). Thus, the production of IL-17 from eosinophils may be important in protection of the host against cancers.

Moreover, since eosinophils produce numerous growth factors such as vascular endothelial growth factor (VEGF), as well as a number of other factors that promote angiogenesis, it is possible that eosinophilic inflammation is implicated in tumor neovascularization (227). Hypothetically, eosinophil-derived cytokines, chemokines, and growth factors may be involved in enhancing T cell-mediated tumor killing, particularly at the level of the local tissue environment where large numbers of infiltrating eosinophils accumulate and are actively degranulating onto tumor cells.

Taken together, these findings suggest that eosinophils may serve as important components of natural immunity, and their cytokines, chemokines, and growth factors may contribute to augment inflammatory responses in allergy and other conditions. Further studies are awaited to understand the involvement of eosinophil-derived immunomodulatory factors in the regulation of allergic inflammation and other inflammatory conditions, as well as the extent to which the release of these factors may be manipulated for therapeutic benefit.

## Conflict of Interest Statement

The authors declare that the research was conducted in the absence of any commercial or financial relationships that could be construed as a potential conflict of interest.
